# Phantom epistasis through the lens of genealogies

**DOI:** 10.1093/genetics/iyaf184

**Published:** 2025-09-05

**Authors:** Anastasia Ignatieva, Lino A F Ferreira

**Affiliations:** Department of Statistics, University of Oxford, Oxford OX1 3LB, United Kingdom; Department of Statistics, University of Oxford, Oxford OX1 3LB, United Kingdom

**Keywords:** gene-by-gene interactions, phantom epistasis, ancestral recombination graphs, genome-wide association studies

## Abstract

Phantom epistasis arises when, in the course of testing for gene-by-gene interactions, the omission of a causal variant with a purely additive effect on the phenotype causes the spurious inference of a significant interaction between two single-nucleotide polymorphisms (SNPs). This is more likely to arise when the two SNPs are in relatively close proximity, so while true epistasis between nearby variants could be commonplace, in practice there is no reliable way of telling apart true epistatic signals from false positives. By considering the causes of phantom epistasis from a genealogy-based perspective, we leverage the rich information contained within reconstructed genealogies (in the form of ancestral recombination graphs) to address this problem. We propose a novel method for explicitly quantifying the genealogical evidence that a given pairwise interaction is the result of phantom epistasis, which can be applied to pairs of SNPs regardless of the genetic distance between them. Our method uses only publicly available data and so does not require access to the phenotypes and genotypes used for detecting interactions. Using simulations, we show that the method has excellent performance at even low distances (around 0.5 Mb), and demonstrate its power to detect phantom epistasis using real data from previous studies. This opens up the exciting possibility of distinguishing spurious interactions in *cis* from those reflecting real biological effects.

## Introduction

The study of the genetic basis of human traits and diseases has progressed rapidly in recent years. Fueled by large biobanks, genome-wide association studies (GWASs) have led to the discovery of thousands of genetic variants associated with a wide array of phenotypes (Tam et al. 2019; Sollis et al. 2023), shedding light on their genetic architecture (Watanabe et al. 2019; Lappalainen et al. 2024) and enabling the prediction of disease risk based on genetic data through polygenic scores (PGSs) (Torkamani et al. 2018; Lambert et al. 2021).

The overwhelming majority of genetic associations identified so far involve variation at a single locus (Tam et al. 2019). Comparatively less attention has been given to gene-by-gene (G×G) interactions, or epistasis, between pairs or larger groups of mutations, where the simultaneous presence of mutations at two or more distinct loci has a combined average effect on the phenotype that is different from the sum of their individual effects in isolation (Phillips 2008; Cordell 2009). It is essential to differentiate functional epistasis, which describes the underlying mechanistic interactions between genes at a biochemical or cellular level, from the quantitative genetic notion of statistical epistasis. The latter, at a population level, measures the deviation from additivity in a statistical model of allelic effects on a phenotype. The magnitude of this statistical epistasis, and the resulting epistatic variance component, is fundamentally dependent on the frequencies of the interacting alleles within the population. Consequently, functional epistasis can be both strong and widespread, yet contribute very little to the total genetic variance if the variants (or, more precisely, the haplotypes carrying the interacting combination of alleles) are rare; this problem is exacerbated by the fact that the frequency of an interaction is dependent on the product of the frequencies of the individual variants, making it significantly lower than either frequency. Moreover, estimates of the additive effect of variants will tend to capture part of the effects of their possible interactions due to standard properties of linear regression. Low epistatic variance in a population therefore does not preclude the existence of significant functional gene interactions. In humans, few signals of statistical epistasis have been found (Wei et al. 2014; Lappalainen et al. 2024) and it has been established that this has a limited role in explaining phenotypic variance (Hill et al. 2008; Sella and Barton 2019; Hivert et al. 2021), and so is unlikely to explain a substantial fraction of “missing heritability” (Manolio et al. 2009) or allow for improved PGS performance. However, detecting a statistical interaction between two parts of the genome suggests a functional interaction between them and so could improve our understanding of the biological function underlying genetic associations (Phillips 2008; Mackay 2014; Domingo et al. 2019), a fundamental open problem in the field (Lappalainen et al. 2024).

The search for genetic interactions is made challenging by the vast search space of possible variant combinations which, for pairwise interactions, grows quadratically in the number of variants considered (Wei et al. 2014). This has raised obstacles along two dimensions. First, exhaustive testing for all possible pairwise combinations of variants has a large computational cost. Second, even if this computational barrier is surmounted, performing a great number of statistical tests requires a stringent multiple testing correction to avoid rampant false positives, resulting in loss of statistical power; this is exacerbated by such tests having a higher-dimensional parameter space than tests for direct effects. Multiple software tools have been developed that make exhaustive testing for epistasis possible (Upton et al. 2016); however they have not yielded much fruit, likely due to a lack of power. As an alternative, methods that first perform a strategic reduction of the search space by leveraging statistical or biological features of the loci under consideration (Sun et al. 2014) appear more promising and have enabled the identification of a few robust interactions (Genetic Analysis of Psoriasis Consortium & the Wellcome Trust Case Control Consortium 2 2010; The Australo-Anglo-American Spondyloarthritis Consortium TASC et al. 2011; Masuda et al. 2015; Lyon et al. 2022).

Recently, a novel statistical challenge to the reliable detection of interactions has been recognized and explored. Termed “phantom epistasis,” this phenomenon arises when the statistical evidence for the existence of a genetic interaction fades when the *additive* effect of a third variant is considered. Phantom epistasis was first clearly identified as a problem following a study which examined possible interactions impacting transcription levels in humans: Hemani et al. (2014, retracted) considered the expression of 7,339 genes, measured through RNA sequencing, and detected 30 statistically significant interactions affecting 19 different genes that replicated in two external datasets. However, Wood et al. (2014) showed with different data that none of these interactions remained statistically significant once the most strongly associated variant with the corresponding phenotype was included in the model. A crucial difference between the datasets used in the two studies was sequencing depth: the former study used genotyping array data (∼530,000 single-nucleotide polymorphisms, SNPs) while the latter used a whole-genome sequencing (WGS) dataset. Wood et al.’s findings suggest that the interactions originally reported were, in effect, tagging a third variant whose effect on the phenotype was fully additive (and which was not included in the data of Hemani et al.).

More recently, de los Campos et al. (2019) have demonstrated, using a simplified statistical model of genetic interactions, that phantom epistasis can arise as the result of mutual but imperfect linkage disequilibrium (LD) between the two spuriously interacting SNPs and a third, unobserved, causal quantitative trait locus (QTL) which has a fully additive effect. They state that it is not possible to empirically test for this condition, as the QTL genotype is unknown. This phenomenon is a direct statistical consequence of third-order LD, the nonrandom association of alleles at three loci (Bennett 1954). In this scenario, the interaction term becomes a proxy for the unobserved causal variant due to this higher-order dependency. The potential for this to create phantom epistasis is not merely theoretical: for instance, Zan et al. (2018) found higher-order LD to be prevalent in *Arabidopsis thaliana* sequencing data. Evolutionary forces such as selection can introduce and maintain such higher-order associations (Grote et al. 1998), potentially creating complex signals of phantom epistasis around functionally important genomic regions. Hemani et al. (2021) reproduced spurious interactions in simulations and recommend adjusting for fine-mapped additive effects when testing for epistasis. Thus, the challenge posed by phantom epistasis remains unsolved, with the best solution currently available being to carefully account for observed additive effects, with no guarantee that this will be sufficient to avoid false-positive interactions. Using WGS data does largely resolve the problem of missing causal variants, although not entirely, since this will still miss structural variants and SNPs appearing in repetitive regions which are difficult to sequence.

In this article, we develop a method that can provide evidence against the existence of an unobserved additive effect which could explain a putative interaction. Our key observation is that, while we cannot (by definition) observe a missing genetic variant, it is possible to calculate a proxy genotype with which any such unobserved QTL would have to be highly correlated for phantom epistasis to emerge, and to search for evidence of its presence—or of its absence—within a genealogical framework. We do this by considering the conditions for phantom epistasis in terms of conditions on the existence of clades of samples within the underlying genealogy. For instance, if the set of samples carrying both of the detected interacting SNPs itself forms a clade within the genealogy, and a variant shared by these samples is present but not included in the statistical analysis, the interaction term will act as a proxy for this unobserved additive-effect variant (likely resulting in a false positive in a test for interactions).

We present a computational tool (Spectre: Searching for Phantom EpistatiC interactions using TREes) implementing two methods of searching for evidence of phantom epistasis, which can be applied to summary statistics from association studies conducted using large-scale genotype data, and genealogies (in the form of ancestral recombination graphs, ARGs) reconstructed using a WGS dataset; we use the 1000 Genomes Project (1KGP, 1000 Genomes Project Consortium 2015). We proceed by calculating genotype proxies for potential unobserved causal (additive effect) QTLs, and then (i) searching for clades in the genealogy that can cause phantom epistasis through being in LD with these proxies and (ii) searching for evidence *against* the existence of such problematic clades. We show that, as previously noted, phantom epistasis is relatively likely to arise when the two SNPs are in close proximity. However, our method allows to explicitly test for the presence of phantom epistasis for SNPs in *cis*, without requiring an arbitrary cut-off on the genetic distance between them as has been suggested in the literature. We use simulation studies to demonstrate the effectiveness of these methods and apply them to the interactions identified by Hemani et al. (2014, retracted), which were shown to be false positives by Wood et al. (2014). Our work presents the first practical computational tool for assessing the likelihood that detected genetic interactions are due to phantom epistasis.

Code implementing the methods is available at https://www.github.com/a-ignatieva/spectre.

## Methods

### Overview of ARGs

**Fig. 1.**
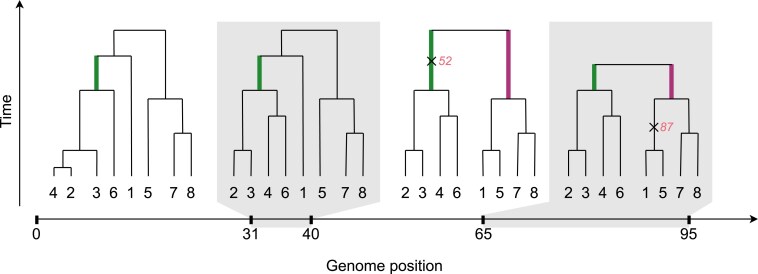
Example of an ARG with n=8 samples, with four local trees shown covering the genomic region [0,95]. The set of samples A={2,3,4,6} forms a clade $\hat{A}$ within the entire interval [0,95] (since there is a branch in each local tree subtending exactly this set of samples, shown in green); this clade is supported by one mutation at position 52 (shown as ×). The set of samples B={1,5,7,8} forms a clade $\hat{B}$ within the interval [40,95] so has a genomic span of 55 bp (in local trees 3 and 4, the branches subtending this clade are shown in purple); this clade is not supported by any mutations. The set C={1,3,4}, for instance, does not form a clade anywhere within the shown region.

The genealogy of a sample of sequences can be fully encoded in the form of an ARG, which can be represented as a series of local trees, describing the local genealogy at each locus (for recent overviews, see for instance Brandt et al. 2024; Lewanski et al. 2024; Wong et al. 2024; Nielsen et al. 2025). Adjacent local trees are highly correlated, as edges tend to persist across multiple local trees before being broken up by recombination. A set of samples can form a clade in the ARG if at some genomic position the set is subtended by a branch of the corresponding local tree. Each clade has a well-defined genomic span, being an interval including all the genomic positions where, in the corresponding local tree, there is a branch subtending exactly the set of samples in the clade. This is illustrated in Fig. 1. ARGs reconstructed from a given sequencing dataset thus provide not only an estimate of the age of each mutation (under the infinite sites model, which we assume throughout), but also an estimate of the genomic span of the clade of its carriers. For any given ARG, it is thus possible to traverse the local trees left-to-right, and record the genomic span of each encountered clade of samples.

Each clade and mutation within the ARG uniquely defines a corresponding set of samples (those belonging to the clade, or carrying the mutation, respectively). A given clade $\hat{Z}$ containing the set of samples *Z*, in turn, defines a corresponding (haploid) genotype vector $z=(z_{1},\ldots ,z_{N})$, with $z_{i}=1$ if i∈Z and $z_{i}=0$ otherwise. Likewise, a given genotype vector v uniquely defines a corresponding set of samples $V=\{i\;:\;v_{i}=1\}$, but it is not necessarily the case that this set forms a clade $\hat{V}$ within the ARG.

### Methods for detecting epistasis

The issue of phantom epistasis concerns only *statistical epistasis*, that is, statistical interaction terms between genetic variables in parametric linear models of phenotypic architecture (Phillips 2008). By construction, these effects are measured while averaging over all other genotypes in a population. There are two widely used models of phenotypic architecture that include epistatic effects. Consider first the simplest linear model of a quantitative phenotype y with a single genetic interaction between two loci (with genotype vectors $x_{1}$ and $x_{2}$):


(1) $$y_{i}=\beta_{1}x_{1i}+\beta_{2}x_{2i}+\beta_{12}x_{1i}x_{2i},$$

where *i* indexes the samples. The canonical test for a genetic interaction is to fit a linear regression model and perform a *t*-test of significance for the estimated $\beta_{12}$ (Cordell 2009) (using logistic regression when the phenotype is binary). A less parsimonious model separates additive from dominance effects and allows interactions between these two classes of effects:


$$\begin{array}{rl} y_{i} & =\beta_{1}x_{1i}+\beta_{1d}I_{x_{1i}}+\beta_{2}x_{2i}+\beta_{2d}I_{x_{2i}}+\beta_{12}x_{1i}x_{2i}+\\ & \;+\beta_{1d2}I_{x_{1i}}x_{2i}+\beta_{12d}x_{1i}I_{x_{2i}}+\beta_{1d2d}I_{x_{1i}}I_{x_{2i}}, \end{array}$$

where $I_{x}=1$ if x=1 and 0 otherwise. Under this model, we can test for the existence of an epistatic effect of any of these four types (additive-by-additive, additive-by-dominance, etc.) at once through an *F*-test with four degrees of freedom.

In their original analysis of epistasis affecting expression levels, Hemani et al. (2014, retracted) performed *F*-tests with four degrees of freedom using the computational implementation of Hemani et al. (2011). In later work, they showed through simulations that such a test is prone to phantom epistasis and stated that the same is true of the *t*-test approach (Hemani et al. 2021), which is the model de los Campos et al. (2019) considered in their work. Given the high-dimensionality of the search space and the fact that very few interactions have been identified using human genetic data, we explore the issue of phantom epistasis using the simpler model (1). This approach makes tractable the derivation of a key result used in our method.

### Phantom epistasis

Consider testing for an interaction between SNP1 and SNP2 (at positions $m_{1}$ and $m_{2}$, with genotype vectors $x_{1}$ and $x_{2}$, and sample sets $X_{1}$ and $X_{2}$, respectively) within the linear regression model


(2) $$y_{i}=\beta_{1}x_{1i}+\beta_{2}x_{2i}+\beta_{12}x_{1i}x_{2i}+\varepsilon_{i},$$

where $\varepsilon_{i}$ is an error term. Assuming that neither the SNPs nor their interaction has a causal effect on the phenotype (such that $\beta_{1}=\beta_{2}=\beta_{12}=0$), it may nevertheless be the case that our estimate of the coefficient $\beta_{12}$ is statistically significant if a third SNP $z_{i}$ which does have a causal effect on $y_{i}$ is correlated with the interaction term after controlling for $x_{1i}$ and $x_{2i}$ (strictly, if the partial covariance of $z_{i}$ and the interaction term conditional on $x_{1i}$ and $x_{2i}$ is sufficiently large relative to the partial variance of the interaction term). This follows from standard linear regression results and was first shown by de los Campos et al. (2019); we provide a detailed derivation in Supplementary Methods, Section S1.3. We note that the same principles of LD with an unobserved causal variant giving rise to phantom epistasis can also cause phantom dominance. Specifically, a spurious dominance effect can be detected at a marker locus if it is in LD with a purely additive causal variant that has a different allele frequency.

Comparing this setting to a standard GWAS where the individual effects of SNPs on a phenotype are tested, there is both a similarity and an important difference worth noting. Whenever we test for the direct effect of a variant on a phenotype, it is understood that a statistically significant effect does not necessarily imply that the variant in question has a causal effect on the phenotype. Rather, we recognize that it may be merely correlated with such a causal effect in its vicinity, a phenomenon known as “tagging,” and that fine-mapping is required to attempt to pinpoint the true causal variant. In a sense, a similar claim could be made about a statistically significant interaction. The crucial difference is that, if a third variant with an *additive* effect explains an apparent interaction signal, this changes the nature of the putative interaction effect being reported. The claim that a statistical interaction has an effect on a phenotype implies that this effect cannot be fully accounted for by any combination of simple additive effects, and that a fundamentally more complex phenomenon is at work (in other words, a purely additive model is not a fully accurate statistical representation of the genetic basis of this phenotype). If it turns out that the additive effect of a third variant can explain the interaction signal, this is no longer true.

To begin examining this issue from a genealogy-based perspective, let us assume a haploid model for simplicity and consider the following example matrix of genotypes:


$$\begin{array}{cccc} x_{1} & x_{2} & s & z\\ 0 & 0 & 0 & 0\\ 1 & 0 & 0 & 0\\ 1 & 1 & 1 & 1\\ 1 & 0 & 0 & 0\\ 0 & 0 & 0 & 0\\ 1 & 1 & 1 & 1\\ 0 & 1 & 0 & 0\\ 0 & 1 & 0 & 0 \end{array}$$

where $s\;:=x_{1}\circ x_{2}$ (“°” denoting element-wise product) represents the simultaneous occurrence of the two mutations, and z is a third mutation that happens to be perfectly correlated with s. If z is the true causal variant for the phenotype but only $x_{1}$, $x_{2}$, and s are included in the model, s will likely appear statistically significant as it is perfectly correlated with the true (additive) causal effect, when in fact no epistasis is present.

**Fig. 2.**
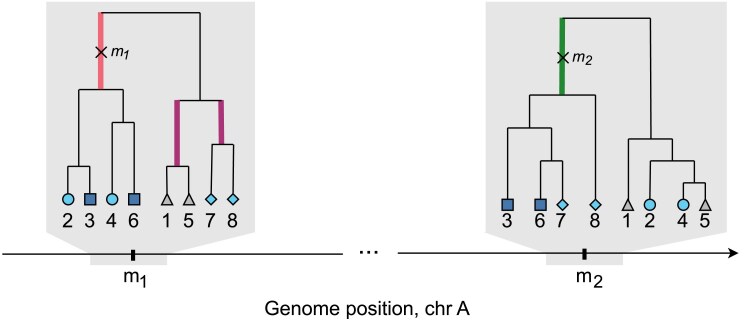
The two mutation events, SNP1 at $m_{1}$ and SNP2 at $m_{2}$, are shown as × on the corresponding branches (in red and green) of the local trees at $m_{1}$ and $m_{2}$, respectively. Here $X_{1}=\{2,3,4,6\}$, $X_{2}=\{3,6,7,8\}$, and the corresponding target sets are S={3,6} (dark blue squares), $K_{1}=\{2,4\}$ (light blue circles), $K_{2}=\{7,8\}$ (light blue diamonds), O={1,5} (grey triangles). In these two trees the set *S* does not form a clade, since there is no branch that subtends only the samples in *S*; the same is true for set $K_{1}$. The sets $K_{2}$ and *O* form clades in the local tree at $m_{1}$; branches subtending these clades are shown in purple. Thus, if the causal mutation occurs on one of the purple branches but is not sequenced, phantom epistasis could arise and result in a significant interaction term between $x_{1}$ and $x_{2}$.

We note that variants like z are not the only kind that can lead to spurious interactions. Suppose that a significant interaction is detected between $x_{1}$ and $x_{2}$. Then define the four “target sets” as


$$\begin{array}{rl} S & =\{i\;:\;i\in X_{1}\text{ and }i\in X_{2}\},\\ K_{1} & =\{i\;:\;i\in X_{1}\text{ and }i\notin X_{2}\},\\ K_{2} & =\{i\;:\;i\notin X_{1}\text{ and }i\in X_{2}\},\\ O & =\{i\;:\;i\notin X_{1}\text{ and }i\notin X_{2}\}, \end{array}$$

where $X_{1}=\{i\;:\;x_{1i}=1\}$ and $X_{2}=\{i\;:\;x_{2i}=1\}$. Phantom epistasis can arise if any of these sets forms a clade in the ARG (at *any* position along the genome), but no SNP corresponding to this clade is included in the model (implying that a causal variant shared by the samples in this clade might exist but has not been sequenced). This is because inclusion of such a SNP would mean that the interaction term can be represented by a linear combination of $x_{1}$, $x_{2}$, and the genotype vector of this SNP. For instance, consider the following matrix corresponding to the ARG in Fig. 2:


$$\begin{array}{cccccc} x_{1} & x_{2} & s & k_{1} & k_{2} & o\\ 0 & 0 & 0 & 0 & 0 & 1\\ 1 & 0 & 0 & 1 & 0 & 0\\ 1 & 1 & 1 & 0 & 0 & 0\\ 1 & 0 & 0 & 1 & 0 & 0\\ 0 & 0 & 0 & 0 & 0 & 1\\ 1 & 1 & 1 & 0 & 0 & 0\\ 0 & 1 & 0 & 0 & 1 & 0\\ 0 & 1 & 0 & 0 & 1 & 0 \end{array}$$

The interaction term can be written equivalently as


$$x_{1}\circ x_{2}=s=x_{1}-k_{1}=x_{2}-k_{2}=x_{1}+x_{2}+o-1$$

(where 1 is a vector of 1’s), so inclusion of any of $s,k_{1},k_{2},o$ would result in the model being unidentifiable. On the other hand, if the genotype of the true causal variant is highly correlated with $s,k_{1},k_{2}$, or o conditional on $x_{1},x_{2}$, omitting this variant from the model will cause spurious inference of a significant interaction effect.

Phantom epistasis can thus arise with the existence of any clade $\hat{Z}$ such that the corresponding set of samples *Z* is highly enough correlated (positively or negatively) with a target set, after conditioning on $x_{1}$ and $x_{2}$. It is important to note that while these four target sets are linearly dependent once the main effects are accounted for (meaning the statistical test for the interaction itself is 1-dimensional), we consider each one as a distinct genealogical scenario. A phantom signal could arise from an unobserved causal variant being correlated with the specific haplotype partition corresponding to any of these sets, so we check all four possibilities.

Note that, theoretically, it does not matter where along the genome $\hat{Z}$ arises. Under the neutral panmictic coalescent model, the probability that a set of |Z| samples forms a clade in a random local tree of size *n* can be easily calculated (Supplementary Methods, Section S1.2); this is negligible unless |Z| is small, or the required level of correlation with a target set is low. The probability of $\hat{Z}$ existing is, however, generally elevated at genomic positions near $m_{1}$ and near $m_{2}$: for instance, since $S=X_{1}\cap X_{2}$ is a subset of $X_{1}$, near $m_{1}$ we need to instead consider the probability that *Z* forms a sub-clade of a clade of size $\left|X_{1}\right|$, which is higher than the probability that *Z* forms a clade in a tree of size *n*. Note that this is true even when $m_{1}$ and $m_{2}$ are very far apart. When $m_{1}$ and $m_{2}$ are close together, however, we know that *S* is a subset of $X_{1}$ and a subset of $X_{2}$, which both form clades in the region $\left[m_{1},m_{2}\right]$, so the probability of interest will be further elevated. This reasoning aligns with phantom epistasis being more likely to affect SNPs nearby in *cis*; it further implies that, for each target set, we only need to consider the probability of a highly correlated clade existing at genomic positions near $m_{1}$ and $m_{2}$.

We note that population structure, through its effects on shared ancestry across the genome, makes it more likely that a specific set of samples (corresponding to a subpopulation) will form a clade at any given locus. The resulting nongametic phase disequilibrium at large distances can be of the same order of magnitude as gametic phase disequilibrium, and both can theoretically cause phantom epistasis. However, the two standard approaches to controlling for population stratification—incorporating principal components of the genetic relationship matrix as covariates in the model or employing linear mixed models—take both of these sources of long-range correlation into account, and are expected to correct for them to a large extent as they attempt to control for the underlying ancestral confounding that generates them. Our method assumes that these corrections have been successfully applied and therefore focuses instead on analyzing the remaining localized signals between variants nearby on the same chromosome.

### Genealogy-based testing for phantom epistasis

Typically, interaction tests are performed using large (unphased) genetic datasets, and hence it is difficult to calculate the target sets for each pair of tested SNPs as above, and not possible to directly estimate the probability that they form clades. We thus take the following approach. For each pair of SNPs with a significant interaction term in an association study, we calculate the corresponding target sets using an ARG reconstructed from WGS data from the same population as (but not necessarily containing samples from) the study cohort from interaction testing. We define each clade by its set of descendant samples, and traverse the ARG, recording the genomic position at which each observed clade first arose, and that at which it disappeared (due to recombination).

We propose two complementary tests to assess the chance of phantom epistasis, each providing a different type of evidence. The two tests are designed to work in tandem: one actively searches for positive evidence of a phantom effect, while the other quantifies the evidence against one. Our first test (Test 1) is a direct search for a potential causal variant. This searches for specific clades in the ARG that are sufficiently correlated with the interaction’s target sets to be a plausible driver of the observed signal. This test is most powerful when it identifies a specific, high-confidence candidate clade (or clades) that could explain away the interaction. However, a failure to find a causal clade with Test 1 is not definitive proof that the interaction is real. Therefore, our second test (Test 2) approaches the problem from the opposite direction by quantifying the residual uncertainty. It evaluates the local genealogy to identify regions where the existence of a phantom epistasis–causing variant cannot be confidently ruled out. The two tests are therefore complementary. The strongest evidence for phantom epistasis arises when Test 1 identifies specific candidate clades that reside within the regions found by Test 2. Conversely, the strongest evidence against phantom epistasis (and thus in favor of a true biological interaction) is when Test 1 finds no plausible candidate clades and Test 2 does not find regions where phantom epistasis is likely to arise.

#### Test 1: Searching for additive effects not included in the model

Our first method of checking for phantom epistasis is by using the 1KGP ARG to check for clades (within ±1 cM of each SNP) which, if excluded from the regression model, could cause a significant (but phantom) interaction to arise or, from a different perspective, could cause a significant interaction term to cease to be significant if included.

**Fig. 3.**
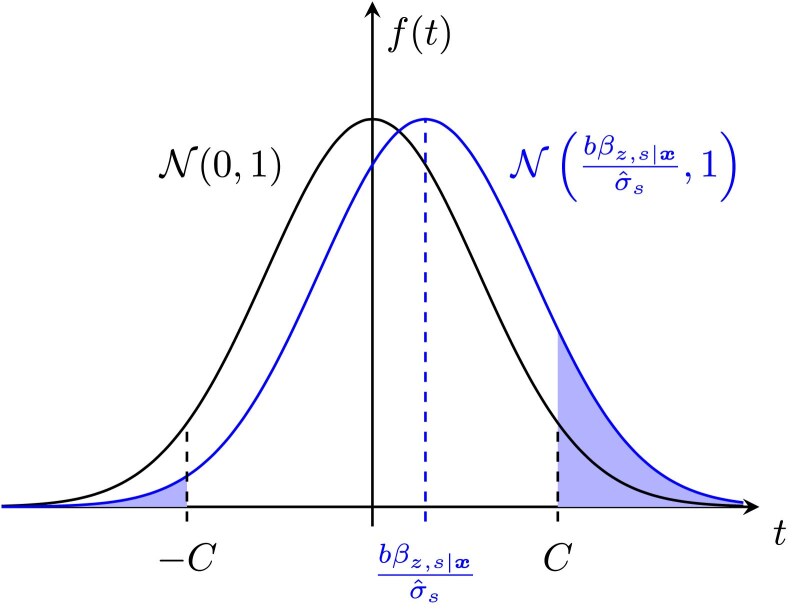
Black curve: standard normal density of test statistic under the null; blue curve: normal density of test statistic in the presence of an omitted variant $z_{i}$, with mean $b\beta_{z,s|x}/{\hat{\sigma }}_{s}$ and unit variance. Shaded area shows probability of incorrectly rejecting the null hypothesis $\beta_{s}=0$ in a two-sided *Z*-test with significance level *α*, where $C\;:=\Phi^{-1}(1-\alpha /2)$. Test 1A calculates *b* which makes the shaded area equal to a set probability *P*.

As detailed in Supplementary Methods, Sections S1.3 and S1.4, we build on the derivations of de los Campos et al. (2019) and on standard results for linear regression to derive the asymptotic distribution of the test statistic of the estimated coefficient ${\hat{\beta }}_{12}$ in the regression model (2) in a statistical test of the null hypothesis $\beta_{12}=0$ (i.e. a standard *Z*-test) under the assumption that there is no interaction but there is a correlated third variant $z_{i}$ with a nonzero effect *b* on the phenotype (*b* is in units of standard deviations of the phenotype). Based on this we can compute, for a given significance level *α* of the original test for interaction (which yields a threshold $C\;:=\Phi^{-1}(1-\alpha /2)$) and an assumed effect *b* of the variant $z_{i}$, the probability that the test statistic exceeds the threshold *C* in absolute value, such that the null hypothesis is (incorrectly) rejected in a two-sided test. Defining an auxiliary variable $s_{i}\;:=x_{1i}x_{2i}$ with corresponding coefficient $\beta_{s}$, this is given by


(3) $$\begin{array}{rl} P & \;:=\text{Pr}\left(\frac{\left|{\hat{\beta }}_{s}\right|}{{\hat{\sigma }}_{s}}\geq C\right)=\Phi \left(-C-\frac{b\beta_{z,s|x}}{{\hat{\sigma }}_{s}}\right)+\left[1-\Phi \left(C-\frac{b\beta_{z,s|x}}{{\hat{\sigma }}_{s}}\right)\right], \end{array}$$

where ${\hat{\beta }}_{s}$ is the estimate of $\beta_{s}$, ${\hat{\sigma }}_{s}\;:=\sqrt{\hat{\text{Var}}({\hat{\beta }}_{s})}$ its standard error and $\beta_{z,s|x}$ the true coefficient of $s_{i}$ in a population regression of $z_{i}$ on $x_{1i},x_{2i}$ and $s_{i}$ (Fig. 3). This last coefficient depends on the partial covariance of $s_{i}$ and $z_{i}$ conditional on $x_{1i},x_{2i}$ and can be readily estimated from the data used to build the ARG. And as we show in Supplementary Methods, Section S1.3.6, ${\hat{\sigma }}_{s}$ can be accurately approximated for polygenic phenotypes using only the genotype data in the same dataset.

However, *b* is fundamentally unknowable in practice as $z_{i}$ is unobserved. So we reframe equation (3) by instead setting a range of probabilities *P* of rejecting the null, and for each clade in the ARG we calculate the corresponding values of *b* that yield exactly those probabilities (with larger absolute values of *b* leading to greater probabilities of rejection). These values are reported by our software to aid the user in assessing how likely a given interaction is to be a false positive: if there is a third variant which need only have a small effect size to produce a large probability of incorrectly rejecting the null, this warrants caution in the confidence attached to that interaction. We refer to this procedure as Test 1A, and the idea is illustrated in Fig. 3.

**Fig. 4.**
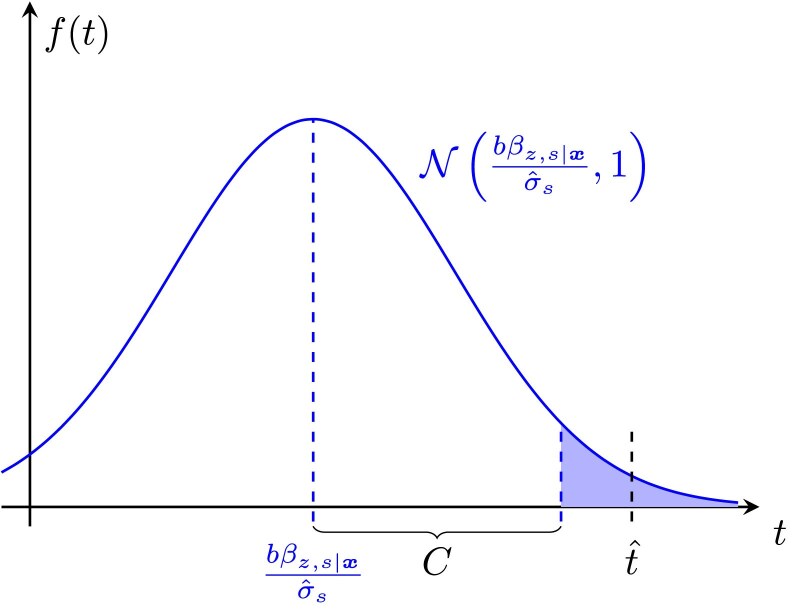
Curve shows density of the test statistic in the presence of an omitted variant; $\hat{t}$ is the value of the test statistic in the original test for interactions; C is the (two-sided) critical value. Test 1B calculates *b* such that $\frac{b\beta_{z,s|x}}{{\hat{\sigma }}_{s}}+C$ aligns with $\hat{t}$.

We also implement an alternative approach based on equation (3). Suppose that there is a third variant $z_{i}$ with effect size *b* leading to bias in the estimated coefficient ${\hat{\beta }}_{s}$ such that this coefficient converges to $b\beta_{z,s|x}$ (Supplementary Methods, Section S1.3.5). We now consider testing the nonzero null hypothesis that $\beta_{s}=b\beta_{z,s|x}$, i.e. testing whether the estimated effect of $s_{i}$ is larger than would be expected if this interaction had no real effect on the phenotype and were only tagging the effect of the unobserved third variant $z_{i}$. For instance, given a positive observed value for the test statistic, and an estimated $\beta_{z,s|x}$ for a certain clade, we compute the value of *b* that would make the test statistic fall exactly at the boundary of the upper rejection region, so that the adjusted null would be rejected (just) in a two-sided test with the same significance level as the original test (Fig. 4). This gives, for each clade, a threshold for *b* above which the interaction would cease to be significant. If this value for *b*, across all clades, is large, we will be more confident in that interaction as it can “survive” the presence of an unobserved variant with an effect up to that size. We refer to this procedure as Test 1B.

#### Test 2: quantifying evidence against existence of unobserved additive effects

While the approach described above will identify observed sequenced variants (and those potentially shared by samples in inferred clades) which can be the cause of spuriously inferred significant interactions, it cannot not completely rule out the possibility that such a variant exists. This can be the case if, for instance, the causal variant is a structural variant, or is present in a repeat-heavy region which is difficult to sequence, or simply due to the unavoidable presence of topological uncertainty in ARG reconstruction (Hayman et al. 2023).

We thus propose a method to consider each clade in the ARG and, within its genomic span, quantify the evidence it provides *against* the possibility of an omitted variant that causes the interaction term to have a significant effect size. To illustrate the basic idea, notice that in Fig. 2 the fact that the set {2,3} forms a clade within the genomic span of the local tree at $m_{1}$ provides evidence against the possibility of the set {3,6} forming a clade within this span. Using similar reasoning, for each target set *G*, we consider in turn each clade $\hat{A}$ of the ARG (within ±1cM of each SNP), and check whether the existence of $\hat{A}$ disproves the existence of all other possible clades $\hat{Z}$ that are highly correlated with *G* and provide a sufficiently high probability of falsely rejecting the null hypothesis (or, conversely, give a sufficiently small *b* yielding such a probability) (Supplementary Methods, Section S1.5). Thus, at each position along the genome, we estimate the minimum value of the effect size that a hidden variant would need to have to result in a significant (phantom) interaction. This is a heuristic procedure since clearly not all possible clades are considered (since there would be an astronomical number of these, for even small ARGs), rather we make a simplifying assumption and only consider possible clades with a high direct correlation with *G*. The point of this procedure (which we call Test 2) is to narrow down the particular regions of the genome where phantom epistasis is more likely.

In summary, the method calculates the minimum (absolute) value of the effect size that a hidden variant would need to have for phantom epistasis to explain the observed data, which can then be compared against a reasonable upper bound for the absolute value of the effect size for the given trait of interest. This is done by using the clades that already exist in the ARG (Tests 1A and 1B), as well as clades that could feasibly exist but might not be present in the single reconstructed ARG being analyzed due to reconstruction uncertainty (Test 2).

## Results

### Simulation studies

We simulated ARGs for 20,503 European individuals using stdpopsim (Adrion et al. 2020) (using the “AmericanAdmixture_4B18” demographic model, the chromosome 22 HapMapII GRCh37 recombination map, and the default mutation rate parameter of $2.36\cdot 10^{-8}$ per bp per generation). For 10,000 of the individuals, we first simulated 100 cases of phantom epistasis. In each case we simulated a quantitative phenotype with one causal variant (true effect size 0.2 and heritability $h^{2}=0.01$), masked this variant, and tested for interactions in the surrounding 400 kb region. We set the significance threshold at 0.05, applying a Bonferroni correction based on the total number of SNP pairs after LD pruning (using Plink (Purcell et al. 2007) with window size 50, step size 5, $r^{2}$ threshold 0.7). For the significant hits (which we successfully replicated using a disjoint sample of 10,000 other individuals from the same simulation) we then ran Spectre and recorded the minimum value of *b* for Tests 1A and 1B (for Test 1A, we set P=0.5). We did this using the simulated ARG for the held-out 503 individuals (the same number of individuals as in the 1KGP ARG subset to European samples), as well as the ARG reconstructed for that data using Relate (Speidel et al. 2019). We repeated this for causal SNPs of varying frequency (from 0.01 to 0.25). Next, with the same data, we simulated true interactions by selecting SNP pairs a distance *R* apart (with R∈{100kb,500kb,5Mb}), such that the frequency of haplotypes carrying both SNPs varied between 0.01 and 0.25. We then simulated a purely epistatic phenotype (interaction effect size 0.2 and heritability $h^{2}=0.01$, 0 main effects for SNP1 and SNP2), carried out regression testing (using a Bonferroni-corrected threshold) and ran Spectre as above. The code used to run the simulations and analyze the output is available at https://www.github.com/a-ignatieva/spectre-paper.

**Fig. 5.**
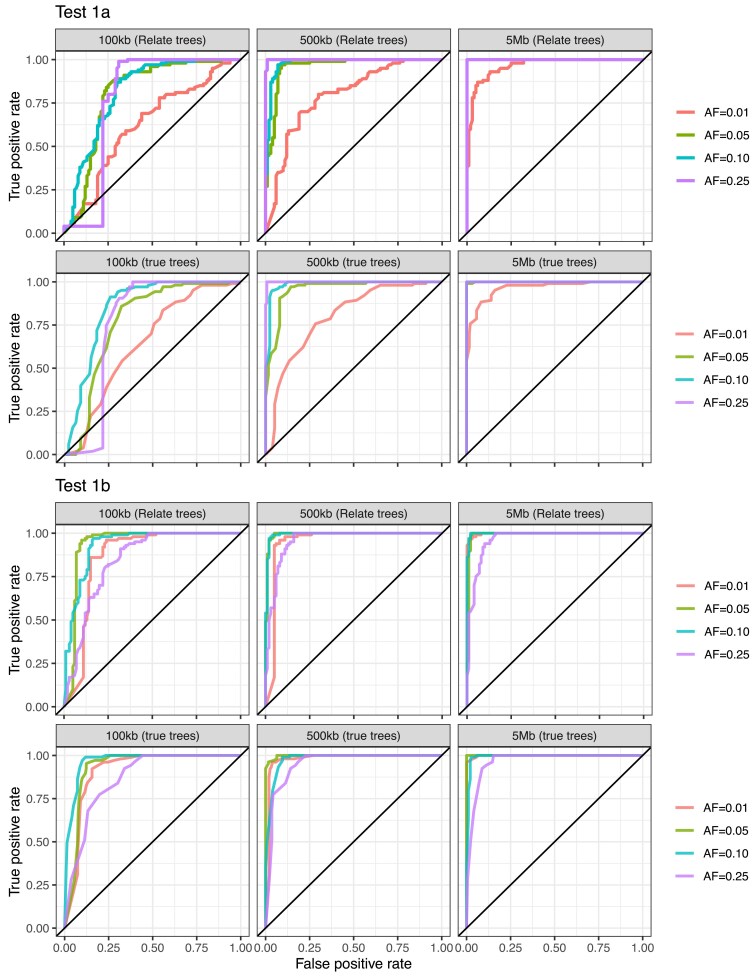
Results using simulated data, with 10,000 individuals for interaction testing, and 503 individuals in the ARG. Top two rows: ROC curve for Spectre results for Test 1A, using ARG reconstructed using Relate (top row) and the simulated ARG (second row). Bottom two rows: ROC curve for Spectre results for Test 1B. For simulations of phantom epistasis, colors show allele frequency of the true causal omitted variant. For simulations of true epistasis, colors show frequency of the overlap between the two SNPs, and columns correspond to different distances between the two interacting SNPs.

Figure 5 shows the resulting ROC curves (obtained by varying the value of the upper bound for a “reasonable” effect size). Both tests demonstrate excellent performance for variants with allele frequency greater than 0.01, and the method can accurately discriminate between true and phantom epistasis for variants only 500 kb apart. We observe a very small reduction in performance for reconstructed versus true ARGs, since the values of *b* calculated using true and Relate ARGs are very close (Supplementary Fig. S3). This is as can be expected, since we only condition on the topology of the ARGs (and not, explicitly, on the event times, which are much more difficult to estimate).

We note that when our method detects phantom epistasis, it is not possible to state in these cases that the significant interaction is a “false positive” as such, but rather just that it is not possible to distinguish whether epistatic or additive effects are present (due to the fundamental unidentifiability of the model when a variant exists whose set of carriers is highly correlated with a target set). As expected, this decays relatively quickly with increasing genetic distance, and (unless the two SNPs have very few carriers, relative to the size of the ARG) the probability of significant clades arising by chance is relatively low even for SNPs that are 0.5 cM apart. If one or both SNPs have relatively low frequency, however, phantom epistasis can be detected even at large distances, through the existence of significant clades by random chance near one of the SNPs. Note also that the allele frequency at which phantom epistasis can be detected depends on the size of the ARG: larger ARGs mean fewer false inferences of phantom epistasis for rare variants.

### Interactions from Hemani et al. (2014)

In order to examine the power of our method applied to real data, we consider the interactions reported by Hemani et al. (2014, retracted), which were detected using data from 846 individuals. These were, through additional sequencing of 450 individuals, shown to be the result of phantom epistasis by Wood et al. (2014). We consider the 12 (out of 17) *cis* interactions that were replicated by Wood et al. at a nominal 0.05 significance threshold (since our method is not designed to discern other causes of false positives, which can be assumed to have resulted in the lack of replication). We used the ARG reconstructed using 1KGP data (Phase 3, GRCh37) with Relate (Speidel et al. 2019). We converted the ARG into tskit format (Kelleher et al. 2016; Wong et al. 2024), and subsetted this to 503 individuals from European populations to match the data used by Hemani et al. (Powell et al. 2012). We only considered SNPs that map uniquely to a branch in the corresponding local tree, and used the HapMapII GRCh37 recombination map to calculate genetic distances. For Spectre, we used 0.05 as the significance threshold and the *p*-values from Wood et al. (2014, Table 1).

**Fig. 6.**
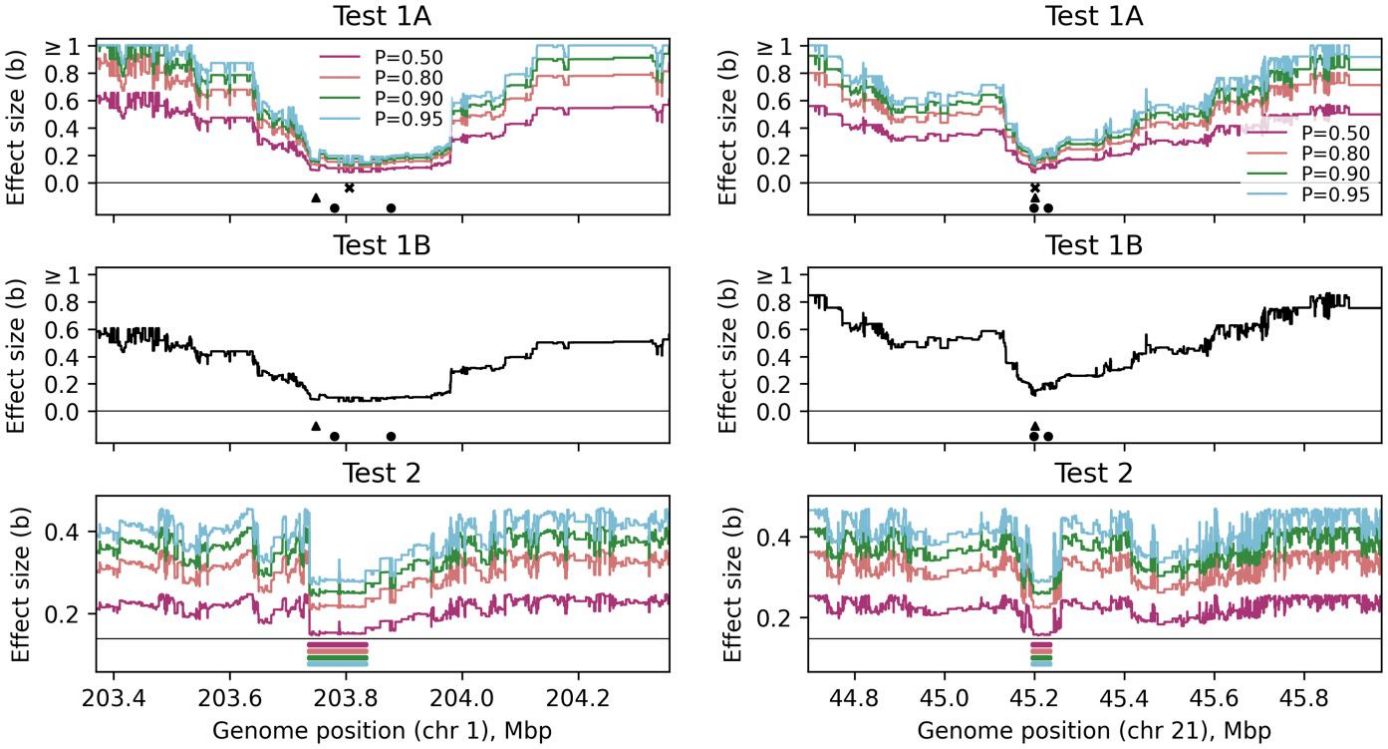
Two examples of results for interactions between SNPs in *cis* reported by Hemani et al. (2014, retracted). The *y*-axis shows the minimum effect size (in units of standard deviations of the phenotype) that an omitted variant would need to have to cause phantom epistasis, based on Tests 1A, 1B, and 2. Colors show different values of the probability *P*, per equation (3); colored horizontal bars for Test 2 show the corresponding regions where the value of *b* lies within 5% of the minimum, highlighting regions where phantom epistasis would be most likely to arise. Black points show positions of SNP1 and SNP2; black cross shows position of clade with the lowest *b* identified by Test 1A and supported by at least one SNP. Position of SNP3 from Wood et al. (2014) is shown as a triangle.

An example of the results for two of the interactions is shown in Fig. 6. The *y*-axis shows the absolute value of the effect size (in units of standard deviation of the phenotype) that an omitted variant would need to have for phantom epistasis to arise, for each of the Spectre tests. At increasing distance away from the two SNPs, this value is relatively high, which suggests that a significant clade is unlikely to exist. In both cases, however, there are regions around the two SNPs (highlighted by Test 2), where the required effect size drops to low values, suggesting that we cannot rule out phantom epistasis. Indeed, in both cases there is a large number of clades (many supported by SNPs) which are highly correlated with one or more of the target sets. A summary of the full results is presented in Table 1. In all cases where the SNPs are nearby in *cis*, the effect sizes are relatively low for each test, suggesting phantom epistasis cannot be ruled out.

**Table 1.** Results for tested interacting SNPs from Hemani et al. (2014, retracted).

SNP1 (Hemani et al.)	SNP2 (Hemani et al.)	Distance (cM)	Test 1A min *b*	Test 1B min *b*	Test 2 min *b*	Top SNP (this paper)
1:203,877,662 (0.4)	1:203,780,591 (0.7)	0.0	0.08	0.07	0.15	1:203,805,538
3:152,234,166 (0.3)	3:152,116,652 (0.5)	0.0	0.10	0.14	0.15	3:152,249,794
11:88,117,962 (0.2)	11:88,077,479 (0.5)	0.0	0.08	0.11	0.15	11:88,070,563
18:74,747,424 (0.5)	18:74,732,087 (0.5)	0.0	0.11	0.00	0.12	18:74,732,472
21:45,230,974 (0.8)	21:45,198,355 (0.7)	0.0	0.07	0.11	0.16	21:45,201,832
8:144,663,661 (0.6)	8:144,613,680 (0.4)	0.1	0.09	0.12	0.14	8:144,608,018
10:76,446,305 (0.7)	10:75,929,517 (0.6)	0.1	0.11	0.08	0.16	10:76,248,814
17:80,890,638 (0.6)	17:80,827,903 (0.6)	0.1	0.07	0.18	0.12	17:80,893,992
19:19,810,050 (0.3)	19:19,738,554 (0.5)	0.1	0.14	0.05	0.16	19:19,672,317
19:36,268,923 (0.4)	19:36,147,315 (0.8)	0.1	0.11	0.15	0.16	19:36,280,047
21:48,027,084 (0.6)	21:47,764,477 (0.2)	0.5	0.10	0.04	0.16	21:47,760,008
21:48,063,862 (0.2)	21:47,776,382 (0.2)	0.6	0.13	0.11	0.18	21:48,068,809

Columns 1 and 2: GRCh37 positions, with frequencies given in brackets. Column 3: distance between the SNPs. Columns 4–6: minimum effect sizes output by Tests 1A, 1B, and 2, respectively. Column 7: SNP giving the lowest value of *b* per Test 1A.

## Discussion

The solutions for addressing phantom epistasis suggested in the literature so far typically involve including fine-mapped variants as covariates in the model, and ignoring pairs of variants that are in close proximity. Our method is the first quantitative approach (to our knowledge) for detecting phantom epistasis, by formulating the issue from the point of view of genealogies, and leveraging the extra information contained in reconstructed ARGs: specifically, inferred clades and their estimated genomic spans. The results can be directly interpreted as a lower bound on the effect size that an omitted variant would need to have in order to cause phantom epistasis (we note that while Test 1B requires summary statistics from the test for interactions, Tests 1A and 2 do not, and can thus be used to screen for pairs of SNPs that can be “safely” tested for interaction for multiple phenotypes).

By deriving the conditions that a clade must satisfy to cause phantom epistasis, de los Campos et al. (2019) showed that it is not necessarily enough to ensure that LD between the two tested SNPs is below some threshold, or that they are far apart in genetic distance, and this is again supported by our results and simulations. Our analysis of previously published interactions from Hemani et al. (2014, retracted) relies on the ARG inferred from the 1KGP data rather than an ARG constructed from samples in the original study. This approach is adopted out of practical necessity, with WGS data required for accurate ARG inference being typically unavailable for most study cohorts. The public 1KGP ARG serves as a high-resolution proxy for the fine-scale genealogical structure and LD patterns of the broader population from which the study samples were drawn. The validity of this cross-dataset approach hinges on the key assumption that the study cohort and the chosen 1KGP samples are well-matched and share demographic history. Under this assumption, the haplotypes (and the clades they form in the ARG) should be highly representative of those in the study cohort. However, this approach has limitations. Differences in population structure or significant genetic drift in the study cohort that is not captured by the 1KGP data could lead to differences in the underlying genealogies, potentially reducing accuracy. Further, differences in allele frequencies between the datasets could alter the composition (and size) of the target sets used for testing. These factors represent potential sources of noise that could reduce the power of our method compared to an analysis using the true, albeit unavailable, ARG which includes the study cohort. Another limitation of our approach, which is constrained by the size of the currently available ARGs, is that the tested SNPs must have relatively high frequency (to allow them to be matched to the SNPs present in the 1KGP ARG). If, for instance, there is only one sequence in the ARG which carries both interacting SNPs, by definition this sequence always forms a clade that is present in the ARG, and our tests will always detect phantom epistasis. Thus, larger ARGs need to be used to test for interactions between rarer variants. This will likely be resolved when ARGs reconstructed for biobank-scale data become available, an area of very active research (e.g. Zhang et al. 2023).

Our framework is built upon ARGs inferred under the infinite-sites model of mutation. This assumption, that every mutation occurs at a new site, is what allows each SNP to be mapped to a unique branch in the genealogy, thereby defining a distinct clade of carriers. This one-to-one mapping between a SNP and a clade is central to our approach. However, the infinite-sites assumption can be violated in reality, particularly at hypermutable sites where recurrent mutations can lead to the same variant appearing independently on different ancestral backgrounds. If the unobserved causal variant responsible for phantom epistasis arose from such recurrent mutations, it would not define a single clade in the ARG; instead, its carriers would be scattered across the genealogy, obscuring the signal our method is designed to detect and potentially reducing its power. This limitation is related to the problem of genotype imputation. The variants most likely to cause phantom epistasis are often those that are absent from genotyping arrays and poorly imputed, which can sometimes be the same variants that violate the infinite-sites model, making both their statistical imputation and their genealogical history difficult to resolve. The efficacy of our method is therefore tied to the fidelity of the underlying genealogical model. Future advances in ARG inference that can accommodate more complex mutational models will enhance the power and accuracy of this approach.

Another limitation is that our analysis is conditioned on a single point estimate of the ARG. This approach does not account for the uncertainty in topologies and clade spans inherent in genealogy reconstruction (Hayman et al. 2023; Ignatieva et al. 2025). A more robust framework would integrate our phantom epistasis tests over a posterior distribution of genealogies, for instance by using samples from a method like ARGweaver (Rasmussen et al. 2014) or SINGER (Deng et al. 2025), to ensure that the conclusions are not driven by artifacts of a single inferred genealogy. We did not pursue that approach here due to methodological and computational constraints. Our method requires ARGs inferred from large sample sizes to capture lower-frequency variants, a scale at which Bayesian ARG reconstruction tools cannot be feasibly applied. Moreover, we found in simulations that our method is very robust to ARG reconstruction error, so the impact of using a single ARG estimate is limited. We also note that within unmappable regions where the ARG cannot be reconstructed our tests cannot provide useful information. However, with the increasing availability of complete long-read sequencing datasets, and future development of genealogy reconstruction methods using such data, our tests could be used to test for phantom epistasis even in such inaccessible regions.

Our approach does not require access to the genetic and phenotypic data used in association testing, only summary statistics. It is important to stress that the method takes as input pairs of SNPs that have been detected to have significant interaction effects. It can thus only specifically detect whether these results are likely to be due to phantom epistasis as defined here (the presence of an omitted additive-effect variant). The method will not differentiate, for instance, false positives due to general inflation of test statistics or other technical issues with the test for interactions. Thus, the method does not remove the necessity of constructing sensitive and powerful tests for detecting epistasis, checking for the inflation of test statistics and replicating the results across multiple datasets. It is also important to distinguish our local fixed-effects modeling approach from the whole-genome random-effects models (e.g. linear mixed models) often used for heritability estimation or controlling for population structure. Our method is designed as a *post hoc* tool to analyze a specific signal, and it intentionally uses a more localized and simpler model to directly assess the hypothesis that the signal is caused by a single nearby unobserved variant. This avoids the complexities of conditioning on the entire genome, which would alter the local covariance structure we investigate. Still regarding the differences between local statistical models and whole-genome approaches, we note that the limited impact of phantom epistasis on the estimation of the phenotypic variance explained by *cis* interactions recently reported in the literature (Smith et al. 2024) was derived under a whole-genome random-effects model. Therefore, the consequences of this phenomenon may be more severe when estimating the fixed effects of only a few variants as is the case in standard epistasis testing.

Our framework operates on phased haplotypes, and we have focused on how gametic phase disequilibrium (LD on a single haplotype) can lead to phantom epistasis. A related issue in diploid organisms is nongametic phase disequilibrium (the correlation between alleles on homologous chromosomes) which can be of the same magnitude as gametic phase disequilibrium at large genetic distances. This phenomenon is primarily a consequence of population structure, which can create spurious associations across the genome. Standard practice in association studies is to mitigate this by including principal components of the genetic relationship matrix as covariates in the model or using linear mixed models. These corrections for population structure are agnostic to whether spurious associations arise from gametic or nongametic disequilibrium, as they aim to control for the underlying ancestral confounding that generates them. Our method is therefore designed as a downstream tool to be applied to signals that have already been adjusted for such broad-scale confounding and, assuming that these corrections were effective, allows us to focus specifically on analyzing whether the remaining signal can be explained by the fine-scale local haplotype structure captured by the ARG.

While our analysis has focused on how LD with a single additive causal variant can generate spurious signals of epistasis between two SNPs, it is important to also consider the reverse scenario. When attempting to identify an epistatic interaction between two causal variants through SNPs that tag them, LD can have the opposite effect of masking the epistatic signal: as demonstrated by Hemani et al. (2013), the statistical power to detect true epistatic effects using markers tends to be more severely compromised by imperfect LD than the power to detect additive effects. This is because the measured epistatic interaction is dependent on the LD between both markers and their respective causal loci, leading to a more rapid decay of the detectable signal as LD weakens. This creates a significant downward bias, increasing the probability that genuine epistatic effects are underestimated or undetected. Together, these two opposing phenomena highlight that LD can not only create false-positive signals of interaction but also obscure true signals, complicating efforts to resolve the genetic architecture of complex traits.

A key strength of our method is that, as noted previously in the literature, phantom epistasis appears likely to arise if the two tested SNPs are in close proximity, and significant interactions between such SNPs are typically assumed to be false positives. In general, true epistasis between nearby SNPs may well be commonplace, and the problem is only that detecting this is very difficult, in part due to the fundamental unidentifiability of the model if a variant exists whose set of carriers is correlated with a target set. Our method explicitly quantifies the genealogical evidence of this, regardless of the distance between the two SNPs, and thus offers a way of distinguishing which interactions between nearby SNPs might represent true biological effects.

An important consideration is the interpretation of clades within the inferred ARG that are not supported by any observed mutations. Intuitively, if a clade lacks supporting variants in a WGS panel like the 1KGP, it might seem unlikely to harbor an unobserved causal variant. However, a clade unsupported by SNPs could still tag a causal structural variant or a variant located in a genomic region that is difficult to sequence or map with short reads. These types of variants are often poorly captured in standard variant call sets but would still generate the genealogical signature our method is designed to assess. Therefore, the presence of such an unsupported but otherwise problematic clade (which our method is tailored to detect) still represents a potential source of phantom epistasis that cannot be dismissed by searching for observed SNPs in a reference panel. This benefit from using the ARG is another advantage of the method. An additional strength is that, since the ARG is a succinct representation of the observed genetic variation, testing clades in the ARG rather than individual SNPs is computationally efficient (and, in the case of Tests 1A and 2, can be performed only once for a genetic dataset and yield results that are applicable to many phenotypes).

The use of WGS data and dense imputation almost resolves the challenge of phantom epistasis by ensuring that most additive causal variants are either directly genotyped or accurately inferred. This leaves phantom epistasis a concern primarily for variants that are poorly imputed or structurally complex. However, this approach is contingent on the availability of large WGS datasets and imputation panels, which are rarely available for species other than humans. Our method therefore provides a robust (and computationally efficient) alternative for epistasis testing in situations where dense imputation is not feasible, such as studies in nonhuman organisms or when only a relatively small WGS sample is available. While most ARG methods have been tailored for use with human data, this presents a fruitful avenue for future research as availability of sequencing data expands across the tree of life.

## Supplementary Material

iyaf184_Supplementary_Data

## Data Availability

Code implementing the methods is publicly available at https://www.github.com/a-ignatieva/spectre. Code used to run simulations and produce the figures, and the resulting files, are publicly available at https://www.github.com/a-ignatieva/spectre-paper. We used the 1KGP ARG reconstructed using Relate from Speidel et al. (2019). Summary statistics for Hemani et al. (2014, retracted) SNPs were obtained from Table 1 of the replication analysis in Wood et al. (2014). Supplemental material available at GENETICS online.
