# Supplementary material for: Phantom epistasis through the lens of genealogies
Source: Genetics. 2025 Sep 5;232(1):iyaf184. doi: 10.1093/genetics/iyaf184 (PMC12774852; doi:10.1093/genetics/iyaf184)
Supplement: iyaf184_Supplementary_Data [file iyaf184_supplementary_data.pdf]

SUPPLEMENTARY INFORMATION

Phantom epistasis through the lens of genealogies

Anastasia Ignatieva[*,1] and Lino A. F. Ferreira[1]

[1]Department of Statistics, University of Oxford
[*]Correspondence: anastasia.ignatieva@stats.ox.ac.uk

Contents

S1 Supplementary methods **2**
S1.1 Summary of notation 2
S1.2 Probability that a set of samples forms a clade in a local tree 2
S1.3 Statistical conditions for phantom epistasis 3
S1.3.1 The true model 3
S1.3.2 The model we fit 3
S1.3.3 Notation 3
S1.3.4 Assumptions 4
S1.3.5 Asymptotic behaviour of the least squares estimator of $\boldsymbol{\beta}$ 4
S1.3.6 Deriving the probability of false-positive interactions 6
S1.4 Searching for clades correlated with a target set 9
S1.5 Evidence against existence of clades correlated with a target set 9

S2 Supplementary figures **11**

S1 Supplementary methods

S1.1 Summary of notation

Variable	Description
n	Total sample size of the ARG
N	Total sample size used in statistical testing for interactions
A	Set of samples $\{a_1, \ldots, a_k\}$
$\|A\|$	Size of set A, $\|A\| = k$
$\widehat{A}$	Clade in the ARG containing exactly the samples in set A
$\widetilde{\boldsymbol{a}}$	Haploid genotype vector $\widetilde{\boldsymbol{a}} = (\widetilde{a}_1, \ldots, \widetilde{a}_n)$ where $\widetilde{a}_i = 1$ if $i \in A$, 0 otherwise
$\boldsymbol{a}$	Normalised genotype vector $\boldsymbol{a} = \widetilde{\boldsymbol{a}} - \sum_{i=1}^{n} \widetilde{a}_i / n$
$A \cap B$	Intersection set $\{i : i \in A \text{ and } i \in B\}$
X_1, X_2	Sets of samples carrying SNP1 and SNP2, respectively
m_1, m_2	Genomic positions of SNP1 and SNP2, respectively
S	Set of samples corresponding to the interaction term, $S = X_1 \cap X_2$
$\widetilde{\boldsymbol{x}}_1$, $\widetilde{\boldsymbol{x}}_2$, $\widetilde{\boldsymbol{s}}$	Genotype vectors for SNP1, SNP2 and their interaction, with $\widetilde{\boldsymbol{x}}_1 = (\widetilde{x}_{11}, \ldots, \widetilde{x}_{1n})$ and $\widetilde{x}_{1i} = 1$ if $i \in X_1$ and 0 otherwise
$\boldsymbol{x}_1$, $\boldsymbol{x}_2$, $\boldsymbol{s}$	Normalised genotype vectors for SNP1, SNP2 and their interaction
$\boldsymbol{X}$	Genotype matrix $[\boldsymbol{x}_1 \| \boldsymbol{x}_2 \| \boldsymbol{s}]$
σ_1^2, σ_2^2, σ_s^2	Variance of $\boldsymbol{x}_1$, $\boldsymbol{x}_2$ and $\boldsymbol{s}$, respectively
σ_{12}, σ_{1s}, σ_{2s}	Covariance of $\boldsymbol{x}_1$ and $\boldsymbol{x}_2$, $\boldsymbol{x}_1$ and $\boldsymbol{s}$, and $\boldsymbol{x}_2$ and $\boldsymbol{s}$, respectively
$\boldsymbol{y}$	Vector of phenotype values (standardised to have zero mean and unit variance)

Note that, for readibility, in the main text we do not distinguish between unnormalised and normalised genotype vectors (since this does not cause ambiguity in the narrative). Without loss of generality, all variables (including s_i) are assumed to have mean zero. This simplifies derivations as it allows us to omit and ignore intercept terms.

S1.2 Probability that a set of samples forms a clade in a local tree

Consider the probability that the target set S forms a clade in a random coalescent tree with n samples. This requires that the samples in S coalesce together before they coalesce with any of the other samples, giving the recursions

$$p_{i,j} = \frac{\binom{i}{2}}{\binom{i+j}{2}} p_{i-1,j} + \frac{\binom{j}{2}}{\binom{i+j}{2}} p_{i,j-1},$$

where i is the number of lineages currently in the tree subtending S, j is the number of lineages currently in the tree *not* subtending S, and $p_{i,j}$ is the probability that S forms a clade given i and j. The boundary conditions are $p_{1,j} = 1\ \forall j$ and we solve for $p_{|S|,n-|S|}$. This can be solved explicitly:

$$p_{|S|,n-|S|} = \frac{2}{(|S|+1)\binom{n-1}{|S|-1}},$$

see, for instance, Hein et al. (2004) [p. 84, eq. (3.26)]. For large n, unless $|S|$ (or $n - |S|$) is small, this probability is very small.

However, we know that S is a subset of X_1, which forms a clade $\widehat{X}_1$ at position m_1. So the relevant probability of interest is instead $p_{|S|,|X_1|-|S|}$, which is larger than $p_{|S|,n-|S|}$. In fact, in some *region* around m_1, this probability will be elevated (before the local trees are broken up by recombination). The same argument holds for the other target sets. Notice that this does not depend on m_1 and m_2 being close together. However, if m_1 and m_2 *are* close together, the probability that S forms a clade will be much higher, since S a subset of X_1 and a subset of X_2, which both form clades within the region $[m_1, m_2]$.

S1.3 Statistical conditions for phantom epistasis

In practice, it is enough for a set Z which is sufficiently highly correlated with S to form a clade $\widehat{Z}$ in order for phantom epistasis to emerge. We now derive the exact conditions that such a set must satisfy.

S1.3.1 The true model

Suppose that we have data on a sample of N individuals, indexed by i, for whom we observe a quantitative phenotype y_i. We assume the same theoretical setup considered by de los Campos et al. (2019) and so examine the effects of variation at three loci, denoted by z_i, x_{1i} and x_{2i}, on this phenotype. For clarity, we first re-derive the results of de los Campos et al. on the least squares estimator of the interaction coefficient before considering the conditions under which we expect phantom epistasis to occur.

The first variant, z_i, is assumed to be the only 'causal locus': together with a zero-mean error term δ_i with constant variance for all individuals, it completely determines the phenotype:

$$y_i = bz_i + \delta_i. \tag{S1}$$

From this theoretical setup, it is clear that there is no epistasis affecting y_i. However, we assume that the true causal locus z_i is not observed; instead, we only observe x_{1i} and x_{2i}, two variants that may be correlated with z_i.

S1.3.2 The model we fit

We fit the following linear regression model which allows for an epistatic relationship between x_{1i} and x_{2i} with an effect on the phenotype:

$$y_i = \beta_1 x_{1i} + \beta_2 x_{2i} + \beta_{12} x_{1i} x_{2i} + \varepsilon_i.$$

Since the true additive effect z_i is unobserved, fitting the model above may suggest an epistatic relationship between x_{1i} and x_{2i} if the estimate for the coefficient β_{12} is significant.

We will now derive the probability of obtaining a significant estimate for β_{12}, which will depend on the partial covariance of the simultaneous occurrence of variants x_{1i} and x_{2i} (after conditioning on x_{1i} and x_{2i}) and the true additive effect z_i.

S1.3.3 Notation

To simplify notation, we define auxiliary variables $s_i := x_{1i}x_{2i}$ and $\beta_s := \beta_{12}$, and so write the model to be estimated as follows:

$$y_i = \beta_1 x_{1i} + \beta_2 x_{2i} + \beta_s s_i + \varepsilon_i. \tag{S2}$$

To aid with our derivations below, we also write the model in an equivalent matrix formulation:

$$\boldsymbol{y} = \boldsymbol{X}\boldsymbol{\beta} + \boldsymbol{\varepsilon}, \tag{S3}$$

where $\boldsymbol{y} = (y_1, \ldots, y_n)$ (and similarly for $\boldsymbol{\varepsilon}$), the genotype matrix is

$$\boldsymbol{X} = \begin{bmatrix} x_{11} & x_{21} & s_1 \\ x_{12} & x_{22} & s_2 \\ & \cdots & \\ x_{1N} & x_{2N} & s_N \end{bmatrix},$$

and $\boldsymbol{\beta} = (\beta_1, \beta_2, \beta_s)$.

S1.3.4 Assumptions

We make four additional assumptions. First, all variables are independent and identically distributed across individuals. Second, the variances of, and covariances between, all the independent variables x_{1i}, x_{2i}, s_i and z_i exist and are finite. Third, there is no multicollinearity between the variables x_{1i}, x_{2i} and s_i; in other words, the matrix $\boldsymbol{X}$ is full rank. This implies, for instance, that SNP1 and SNP2 are not nested in the genealogy (since then we would have $x_{2i} = s_i$ if SNP2 is on a branch subtended by the branch on which SNP1 is located, for example). Finally, we assume zero correlation between the true error term δ_i and all independent variables in equation (S2) as well as the true causal variant z_i, i.e., $\mathbb{E}(\delta_i z_i) = \mathbb{E}(\delta_i x_{1i}) = \mathbb{E}(\delta_i x_{2i}) = \mathbb{E}(\delta_i s_i) = 0$ for all i.

The second and third assumptions are realistic in genetic applications: Bernoulli or binomial random variables always have finite variances and only non-identical genetic variants that segregate in the sample are considered, guaranteeing no multicollinearity. Note also that, while the model above assumes that the phenotype is influenced by only one genetic locus, we could derive the same results while including an additional polygenic component (e.g., $y_i = b_z z_i + \sum_{p=1}^{P} b_p w_{pi} + \delta_i$) as long as all these additional genetic variants (which would then be part of the error term in the model as it is stated) are uncorrelated with z_i, x_{1i} and x_{2i} so that the fourth assumption holds. The model is therefore more general than it may appear at first, with the key assumption being that there is only one causal genetic locus in the haplotypic block under consideration.

S1.3.5 Asymptotic behaviour of the least squares estimator of $\boldsymbol{\beta}$

We first emphasise that the true causal locus z_i is not part of the set of independent variables included in our regression (as it is not observed), which implies that it is effectively part of the error term. In other words, we may rewrite the model in equation (S2) as follows:

$$y_i = \beta_1 x_{1i} + \beta_2 x_{2i} + \beta_s s_i + (b z_i + \delta_i), \tag{S4}$$

where by assumption we have $\beta_1 = \beta_2 = \beta_s = 0$ and $b z_i + \delta_i = \varepsilon_i$.

From standard results for least squares estimators of linear models (see, e.g., Wooldridge (2010) [§4.2.1]) we know that the presence of a variable in the error term that has a non-zero effect on the dependent variable renders the estimator of the effect of an included regressor inconsistent as long as this variable is correlated with that regressor. It is this fact – that the least squares estimator may not converge to the true value for the parameter of interest β_s, which is zero, even as the sample size becomes infinitely large – that can lead to a false-positive interaction being detected if z_i is correlated with s_i controlling for x_{1i} and x_{2i}. Note that we cannot check empirically whether such correlations are non-zero as we do not observe z_i.

The least squares estimator of the coefficients in the original model as written in (S3), denoted $\hat{\boldsymbol{\beta}} = (\hat{\beta}_1, \hat{\beta}_2, \hat{\beta}_s)$, is computed as follows:

$$\hat{\boldsymbol{\beta}} = (\boldsymbol{X}'\boldsymbol{X})^{-1}\boldsymbol{X}'\boldsymbol{y}.$$

The true model given in equation (S1) is in matrix form $\boldsymbol{y} = b\boldsymbol{z} + \boldsymbol{\delta}$. To aid in our derivations below, we add $\boldsymbol{X\beta}$ (which is zero by assumption) and so have $\boldsymbol{y} = \boldsymbol{X\beta} + b\boldsymbol{z} + \boldsymbol{\delta}$. Plugging in this expression for the true value of $\boldsymbol{y}$ into the formula for the estimator, we get:

$$\begin{aligned}\hat{\boldsymbol{\beta}} &= (\boldsymbol{X}'\boldsymbol{X})^{-1}\boldsymbol{X}'\left(\boldsymbol{X\beta} + b\boldsymbol{z} + \boldsymbol{\delta}\right) \\ &= \boldsymbol{\beta} + b(\boldsymbol{X}'\boldsymbol{X})^{-1}\boldsymbol{X}'\boldsymbol{z} + (\boldsymbol{X}'\boldsymbol{X})^{-1}\boldsymbol{X}'\boldsymbol{\delta}.\end{aligned}$$

We wish to derive the asymptotic behaviour of $\hat{\boldsymbol{\beta}}$. Slutsky's theorem allows us to write its limiting expression as follows (where $\xrightarrow{\text{p}}$ denotes convergence in probability and plim its limit):

$$\hat{\boldsymbol{\beta}} \xrightarrow{\text{p}} \boldsymbol{\beta} + b \times \underset{N\to\infty}{\text{plim}} \left(\frac{\boldsymbol{X}'\boldsymbol{X}}{N}\right)^{-1} \times \underset{N\to\infty}{\text{plim}} \left(\frac{\boldsymbol{X}'\boldsymbol{z}}{N}\right) + \underset{N\to\infty}{\text{plim}} \left(\frac{\boldsymbol{X}'\boldsymbol{X}}{N}\right)^{-1} \times \underset{N\to\infty}{\text{plim}} \left(\frac{\boldsymbol{X}'\boldsymbol{\delta}}{N}\right).$$

We can now apply the weak law of large numbers, which ensures that sample means converge to expectations as $N \to \infty$. Defining a matrix $\boldsymbol{T}$ as follows:

$$\boldsymbol{T} = \begin{bmatrix} \mathbb{E}(x_{1i}^2) & \mathbb{E}(x_{1i}x_{2i}) & \mathbb{E}(x_{1i}s_i) \\ \mathbb{E}(x_{1i}x_{2i}) & \mathbb{E}(x_{2i}^2) & \mathbb{E}(x_{2i}s_i) \\ \mathbb{E}(x_{1i}s_i) & \mathbb{E}(x_{2i}s_i) & \mathbb{E}(s_i^2) \end{bmatrix},$$

we have:

$$\operatorname*{plim}_{N\to\infty} \left(\frac{\boldsymbol{X}'\boldsymbol{X}}{N}\right)^{-1} = \boldsymbol{T}^{-1}.$$

Similarly,

$$\operatorname*{plim}_{N\to\infty} \left(\frac{\boldsymbol{X}'\boldsymbol{z}}{N}\right) = \begin{bmatrix} \mathbb{E}(x_{1i}z_i) \\ \mathbb{E}(x_{2i}z_i) \\ \mathbb{E}(s_i z_i) \end{bmatrix}.$$

And since we assume no correlation between δ_i and x_{1i}, x_{2i}, s_i, we have:

$$\operatorname*{plim}_{N\to\infty} \left(\frac{\boldsymbol{X}'\boldsymbol{\delta}}{N}\right) = \begin{bmatrix} \mathbb{E}(x_{1i}\delta_i) \\ \mathbb{E}(x_{2i}\delta_i) \\ \mathbb{E}(s_i \delta_i) \end{bmatrix} = \mathbf{0}.$$

Plugging in these limits, we obtain:

$$\begin{bmatrix} \hat{\beta}_1 \\ \hat{\beta}_2 \\ \hat{\beta}_s \end{bmatrix} \xrightarrow{\text{p}} \begin{bmatrix} \beta_1 \\ \beta_2 \\ \beta_s \end{bmatrix} + b \times \begin{bmatrix} \mathbb{E}(x_{1i}^2) & \mathbb{E}(x_{1i}x_{2i}) & \mathbb{E}(x_{1i}s_1) \\ \mathbb{E}(x_{1i}x_{2i}) & \mathbb{E}(x_{2i}^2) & \mathbb{E}(x_{2i}s_1) \\ \mathbb{E}(x_{1i}s_1) & \mathbb{E}(x_{2i}s_1) & \mathbb{E}(s_i^2) \end{bmatrix}^{-1} \begin{bmatrix} \mathbb{E}(x_{1i}z_i) \\ \mathbb{E}(x_{2i}z_i) \\ \mathbb{E}(s_i z_i) \end{bmatrix},$$

which is equivalent to equation (3) in de los Campos et al. (2019).

Note that the term multiplying b is by definition the coefficient vector in the following population linear model, or linear projection,[1] of the unobserved variant z_i on the observed variants x_{1i}, x_{2i}, s_i, which we write as:

$$\begin{aligned} z_i &= \gamma_1 x_{1i} + \gamma_2 x_{2i} + \gamma_s s_i + r_i, \quad \text{or} \\ \boldsymbol{z} &= \boldsymbol{X}\boldsymbol{\gamma} + \boldsymbol{r}, \end{aligned} \tag{S5}$$

where r_i are the residuals. The vector of population coefficients in this model is:

$$\boldsymbol{\gamma} = \boldsymbol{T}^{-1} \begin{bmatrix} \mathbb{E}(x_{1i}z_i) \\ \mathbb{E}(x_{2i}z_i) \\ \mathbb{E}(s_i z_i) \end{bmatrix},$$

exactly as above.

Focusing now on the estimator of the coefficient of the interaction term $\hat{\beta}_s$ and noticing the connection with the linear projection, we obtain a well-known result: the limit of the least squares estimator is the true value β_s with the added bias of the true effect of the omitted variant z_i which is weighted by the coefficient of s_i in a multiple regression model of z_i on s_i and all other regressors, in this case x_{1i} and x_{2i}:

$$\hat{\beta}_s \xrightarrow{\text{p}} \beta_s + b\beta_{z,s|\boldsymbol{x}}, \tag{S6}$$

where $\beta_{z,s|\boldsymbol{x}}$ denotes this multiple regression coefficient. This may also be written as:

$$\hat{\beta}_s \xrightarrow{\text{p}} \beta_s + b\frac{\operatorname{Cov}(z_i, s_i | x_{1i}, x_{2i})}{\operatorname{Var}(s_i | x_{1i}, x_{2i})}. \tag{S7}$$

[1] A unique linear projection of this form is guaranteed to exist since the matrix $\boldsymbol{X}$ is non-singular; see, e.g., Wooldridge (2010) [§2.3].

In this example, the interaction s_i has no effect on the phenotype (i.e., $\beta_s = 0$) and therefore the estimate of its effect will be driven solely by the tagged effect of the omitted variable. This estimate will tend to be larger the larger the effect (in absolute size) of the true locus and (loosely) the stronger its correlation with the interaction term of interest after accounting for the main effects.

Writing the linear projection of z_i on x_{1i}, x_{2i}, s_i also enables us to derive two more useful facts about the least squares estimator in this context. Plugging in the formula for z_i in equation (S5) into the regression model in equation (S4), we obtain:

$$\begin{aligned} y_i &= (\beta_1 + b\gamma_1)x_{1i} + (\beta_2 + b\gamma_2)x_{2i} + (\beta_s + b\gamma_s)s_i + (\delta_i + br_i), \quad \text{or} \\ \boldsymbol{y} &= \boldsymbol{X}(\boldsymbol{\beta} + b\boldsymbol{\gamma}) + \boldsymbol{\delta} + b\boldsymbol{r}. \end{aligned}$$

This rewritten model satisfies standard assumptions for least squares estimators: $\boldsymbol{X}$ is full rank and the error term (comprised of δ_i and br_i, where b is a constant) is uncorrelated with all regressors and has constant variance. Therefore we can directly invoke known results (see, e.g., Wooldridge (2010) [p. 59, Theorem 4.2]) to establish the asymptotic Normality of the estimator:

$$\hat{\boldsymbol{\beta}} \overset{a}{\sim} \text{Normal}\left(\boldsymbol{\beta} + b\boldsymbol{\gamma}, \text{Var}(\delta_i + br_i)N^{-1}\boldsymbol{T}^{-1}\right)$$

and the consistency of the usual estimator of the variance of $\hat{\boldsymbol{\beta}}$:

$$\widehat{\text{Var}}(\hat{\boldsymbol{\beta}}) = \frac{\sum_{i=1}^{N} \hat{\varepsilon}_i^2}{N-3}(\boldsymbol{X}'\boldsymbol{X})^{-1} \overset{\text{p}}{\to} \text{Var}(\delta_i + br_i)N^{-1}\boldsymbol{T}^{-1}. \tag{S8}$$

S1.3.6 Deriving the probability of false-positive interactions

Having derived the asymptotic behaviour of both the estimator of the coefficient $\hat{\beta}_s$ and its standard error, we now consider the asymptotic behaviour of the test statistic that is used to assess a possible effect of the interaction term s_i on the phenotype.

A standard t-test of the null hypothesis $\beta_s = 0$ is based on the following test statistic:

$$t\text{-statistic} = \frac{\hat{\beta}_s}{\sqrt{\widehat{\text{Var}}(\hat{\beta}_s)}},$$

which for large samples can be compared to a chosen quantile of the standard Normal distribution. For a chosen level of significance α, and defining $\hat{\sigma}_s := \sqrt{\widehat{\text{Var}}(\hat{\beta}_s)}$ for conciseness, we reject the null hypothesis in a two-sided test if and only if:

$$\frac{|\hat{\beta}_s|}{\hat{\sigma}_s} \geq \Phi^{-1}(1 - \alpha/2) =: C, \tag{S9}$$

where $\Phi(\cdot)$ is the cumulative distribution function of the standard Normal.

The question we seek to answer is: for an interaction term s_i with no effect on the phenotype but which tags an unobserved third variant z_i with a non-zero effect b, under what conditions (on the effect size of z_i and on the relationship between s_i and z_i) do we expect to *incorrectly* reject the null hypothesis of no effect for s_i? This hinges on whether the inequality in equation (S9) is satisfied.

We have seen that $\hat{\beta}_s$ is asymptotically Normally distributed with mean $b\beta_{z,s|\boldsymbol{x}}$ and a variance which is estimated consistently through the usual approach, which implies that the test statistic will be asymptotically Normal with mean $b\beta_{z,s|\boldsymbol{x}}/\hat{\sigma}_s$ and unit variance. The probability P of incorrectly rejecting the null hypothesis is then equal to the probability that a random draw from such a Normal distribution exceeds the significance threshold C:

$$P = \Phi\left(-C - \frac{b\beta_{z,s|\boldsymbol{x}}}{\hat{\sigma}_s}\right) + \left[1 - \Phi\left(C - \frac{b\beta_{z,s|\boldsymbol{x}}}{\hat{\sigma}_s}\right)\right], \tag{S10}$$

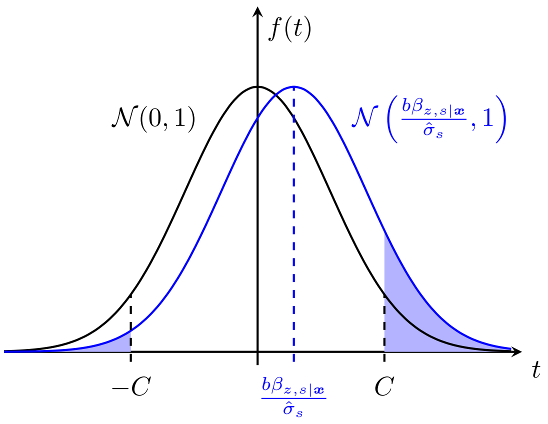

(a) A third variant z with non-zero effect b on the phenotype and which is correlated with the interaction s after accounting for x_1, x_2 causes the test statistic to have an asymptotically Normal distribution with mean $b\beta_{z,s|\boldsymbol{x}}/\hat{\sigma}_s$ and unit variance (probability density function shown in blue; contrast with standard Normal density shown in black). The probability of incorrectly rejecting the null hypothesis $\beta_s = 0$ in a two-sided test with significance level α is given by the shaded area, where we have defined $C \coloneqq \Phi^{-1}(1-\alpha/2)$.

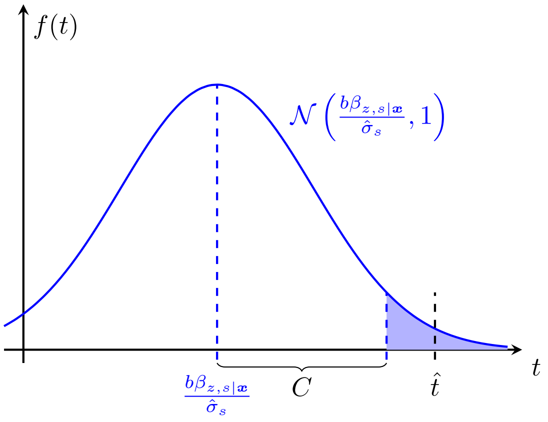

(b) An alternative approach is to perform a two-sided test of the non-zero null hypothesis $\beta_s = b\beta_{z,s|\boldsymbol{x}}$ and thus take into account the bias introduced by a hypothetical third variant z with (additive) effect b. Given an observed test statistic $\hat{t} > 0$, we check whether it falls in the upper rejection region of the shifted distribution such that the null hypothesis is still rejected, i.e., if $\hat{t} \geq b\beta_{z,s|\boldsymbol{x}}/\hat{\sigma}_s + C$. The significance level α is that of the original interaction test and $C \coloneqq \Phi^{-1}(1-\alpha/2)$ as above. For $\hat{t} < 0$, the corresponding plot would be the mirror image and we would assess instead whether the test statistic falls in the lower rejection region.

Figure S1: Two alternative approaches to assessing the reliability of interaction signals given the possible presence of a third variant with non-zero effect on the phenotype.

where the first term on the right-hand side corresponds to the shaded area on the left in Figure S1a and the second term to the area on the right in the Figure.

Equation (S10) has four variables (C, $\beta_{z,s|\boldsymbol{x}}$, $\hat{\sigma}_s$ and b) which together determine the probability of interest P. Conversely, fixing the probability P and three of these variables yields a value for the fourth one. In practice, C is determined by the significance level of the original interaction test whose results we assess and is therefore given. Moreover, $\beta_{z,s|\boldsymbol{x}}$ can be readily estimated for each variant z under consideration from the external dataset on which the ARG is built.

Turning now to the third variable $\hat{\sigma}_s$, since this is the standard error of the interaction coefficient in the regression model it could also be taken from the summary statistics of the interaction test. However, we propose instead to approximate it in a manner that is both highly accurate and which makes this approach independent of the particular phenotype under analysis (the advantages of this

will be discussed below). From equation (S8), we see that $\hat{\sigma}_s$ is computed as follows:

$$\hat{\sigma}_s = \sqrt{\frac{\sum_{i=1}^{N} \hat{\varepsilon}_i^2}{N-3} \, [(\boldsymbol{X}'\boldsymbol{X})^{-1}]_{33}},$$

where $\left[(\boldsymbol{X}'\boldsymbol{X})^{-1}\right]_{33}$ denotes the bottom right (third row, third column) element of the matrix $(\boldsymbol{X}'\boldsymbol{X})^{-1}$, which corresponds to the regressor s_i. Since the phenotypes on which GWASs are run are generally highly polygenic, with hundreds or even thousands of independent associations each with a very small effect, the three regressors in the model given in equation (S2) will jointly account for only a very small percentage of the variance of the phenotype. This in turn implies that the variance of the residuals $\hat{\varepsilon}_i$ will be approximately equal to the sample variance of the phenotype itself. Therefore we can very accurately approximate $\hat{\sigma}_s$ as follows:

$$\hat{\sigma}_s \approx \sqrt{\widehat{\mathrm{Var}}(y_i) \, [(\boldsymbol{X}'\boldsymbol{X})^{-1}]_{33}},$$

where $\widehat{\mathrm{Var}}(y_i)$ is the sample variance of the phenotype. In the derivations above, we could have assumed without loss of generality that the phenotype had been standardised and so had unit variance, in which case we would have $\widehat{\mathrm{Var}}(y_i) = 1$. However, we need not make this additional assumption. If we simply approximate $\hat{\sigma}_s$ as $\sqrt{[(\boldsymbol{X}'\boldsymbol{X})^{-1}]_{33}}$, the term $\sqrt{\widehat{\mathrm{Var}}(y_i)}$ will implicitly divide b in equation (S10). Therefore the values of b that we will obtain through this equation, as we explain in the next paragraph, will be rescaled so that their units are standard deviations of the phenotype and are thus readily interpretable independently of the scale on which the original phenotype is measured – this is a first advantage of employing this approximation. Note that, like $\beta_{z,s|\boldsymbol{x}}$, $\left[(\boldsymbol{X}'\boldsymbol{X})^{-1}\right]_{33}$ can also be easily computed from the external dataset from which the ARG was constructed.

We have seen how the values for three of the four variables in equation (S10) can be set and now turn to consider the fourth variable b. This variable is fundamentally unknowable as we cannot by definition estimate the effect of an unobserved variant. We therefore propose to set a range of probabilities P of incorrectly rejecting the null and, for each of these probabilities, compute the value of b that exactly yields that probability given C, $\beta_{z,s|\boldsymbol{x}}$ and $\hat{\sigma}_s$ (with larger absolute values of b increasing this probability). One can then assess whether the smallest value that the effect of a potential third variant needs to take to lead to an inferential error with a certain probability is small enough that the existence of such a variant is plausible and so warrants concern.

An alternative approach is to assume that there is a problematic third variant, adjust the null hypothesis to account for the expected bias in the inferred coefficient $\hat{\beta}_s$ (which we derived above) and then test this adjusted hypothesis that $\beta_s = b\beta_{z,s|\boldsymbol{x}}$. Intuitively, if $\hat{\beta}_s$ is very large (in absolute value) then we would still reject the adjusted null if the additive effect of the third variant z and its partial correlation with s are not too strong. We then ask, how large can the bias of the test statistic $b\beta_{z,s|\boldsymbol{x}}/\hat{\sigma}_s$ be such that we still reject this non-zero null hypothesis?

We consider a two-sided test of the null hypothesis $\beta_s = b\beta_{z,s|\boldsymbol{x}}$ against the alternative $\beta_s \neq b\beta_{z,s|\boldsymbol{x}}$ with the same significance level α as the original interaction test. For a positive observed value of the test statistic, which we denote by $\hat{t} > 0$, we check whether this statistic falls in the upper rejection region of such a test, i.e., if $\hat{t} \geq b\beta_{z,s|\boldsymbol{x}}/\hat{\sigma}_s + C$ where $C := \Phi^{-1}(1-\alpha/2)$ as above (Figure S1b). (If instead $\hat{t} < 0$, we would assess whether the test statistic falls in the lower rejection region, i.e., if $\hat{t} \leq b\beta_{z,s|\boldsymbol{x}}/\hat{\sigma}_s - C$.)

In practice, given the significance level of the original test, the estimated coefficient $\beta_{z,s|\boldsymbol{x}}$ and the standard error $\hat{\sigma}_s$ (set to $\sqrt{[(\boldsymbol{X}'\boldsymbol{X})^{-1}]_{33}}$ as before), we compute the value b that makes the boundary of the rejection region exactly equal to the observed test statistic so that the null hypothesis is just rejected. For $\hat{t} > 0$ and $\beta_{z,s|\boldsymbol{x}} > 0$, as well as for $\hat{t} < 0$ and $\beta_{z,s|\boldsymbol{x}} < 0$, this gives a positive upper bound for b, with smaller values moving the rejection region boundary away towards zero. Conversely, for $\hat{t} < 0$ and $\beta_{z,s|\boldsymbol{x}} > 0$, or for $\hat{t} > 0$ and $\beta_{z,s|\boldsymbol{x}} < 0$, we would obtain instead a negative lower bound for b. We can then interpret the results as meaning that the interaction identified

would still be significant in a two-sided hypothesis test with the same significance level as the original test as long as the effect b of the correlated clade satisfies this bound. (By focusing on the rejection region farthest from zero, we only reject the null for observed values of $\hat{\beta}_s$ which are larger in absolute value than the expected bias $b\beta_{z,s|\boldsymbol{x}}$ for this coefficient. If we considered also the lower rejection region, our range of 'allowed' values for b would also include values so large in absolute value that the null hypothesis would be rejected because the observed test statistic was too close to zero, which would be unnatural.)

These two approaches are complementary but distinct as they use different sets of information. The first approach does not use the estimated coefficient $\hat{\beta}_s$ or the resulting test statistic, and is thus independent of the phenotype under analysis (we ensured this was the case by approximating $\hat{\sigma}_s$ without using phenotypic information – this general applicability independent of phenotype is a second advantage of the proposed approximation). It simply asks whether two variants can reliably be tested for the effect of their interaction (on any phenotype, provided that it is highly polygenic – or, more precisely, that the variants in question account for very little phenotypic variance – such that the approximation to $\hat{\sigma}_s$ is accurate) with some confidence that a possible interaction signal would not be caused by a hidden third variant, and indeed can be run prior to interaction testing. Given a genetic dataset for which multiple phenotypes are available, this approach could be applied to all pairs of variants within a certain distance from each other to identify pairs that can be tested for interaction; then in a second step we could test whether each of these pairs of SNPs had a significant interaction for each phenotype separately.

In contrast, the second approach does use the estimated test statistic and so requires interaction testing to be performed first and is phenotype specific. It can be thought of as qualifying the interaction signals by asking how robust these are to the presence of hidden third variants: everything else being equal, stronger signals (with a smaller p-value) will be able to withstand third variants with a larger additive effect; however, this robustness will decrease for variants with a highly-correlated neighbouring clade.

S1.4 Searching for clades correlated with a target set

For each clade $\widehat{Z}$ of the ARG within ±1cM of each SNP in the interaction pair under consideration, we can calculate the corresponding normalised genotype vector $\boldsymbol{z}$ and regress this vector on $\boldsymbol{x}_1, \boldsymbol{x}_2$ and $\boldsymbol{s}$ to obtain $\hat{\beta}_s$ (and repeat this replacing $\boldsymbol{s}$ with each of the other target set genotype vectors). This is the basis for the procedure described in Methods, Section 2.4.1: the relevant value of b that can be problematic as just described is then based on the largest value of $\hat{\beta}_s$ across all clades and target sets.

S1.5 Evidence against existence of clades correlated with a target set

Let S be the set of samples in the ARG corresponding to the interaction genotype vector $\boldsymbol{s}$ (that is, the set of samples that carry both SNP1 and SNP2). Suppose clade $\widehat{A}$ appears in some local tree along the genome, with $|S \cap A| = u$, and let $w = |S| - u$ (as illustrated in Figure S2, top line). We would like to check whether $\widehat{A}$ provides evidence against the existence of another clade $\widehat{Z}$ (within the genomic span of $\widehat{A}$) such that the corresponding normalised genotype vector $\boldsymbol{z}$ would satisfy (S9).

Since, in realistic scenarios, ARG reconstruction is not perfect and there is potentially a large amount of uncertainty about the topology of each local tree, we condition only on the fact that the samples in set A form a clade $\widehat{A}$, that is, we use only the most relevant information provided by the ARG on clade existence and genomic span (rather than conditioning on all of the information contained in the graph, such as mutation ages). Then there are six possible scenarios to consider which maximise the correlation between a possible clade $\widehat{Z}$ and the set S, shown in Figure S2. Cases 1–3 (resp. 4–6) correspond to maximising the positive (resp. negative) correlation between Z and S, when $\widehat{Z}$ is a subclade (Cases 1 and 4), superclade (Cases 3 and 6) or disjoint from $\widehat{A}$ (Cases 2 and 5).

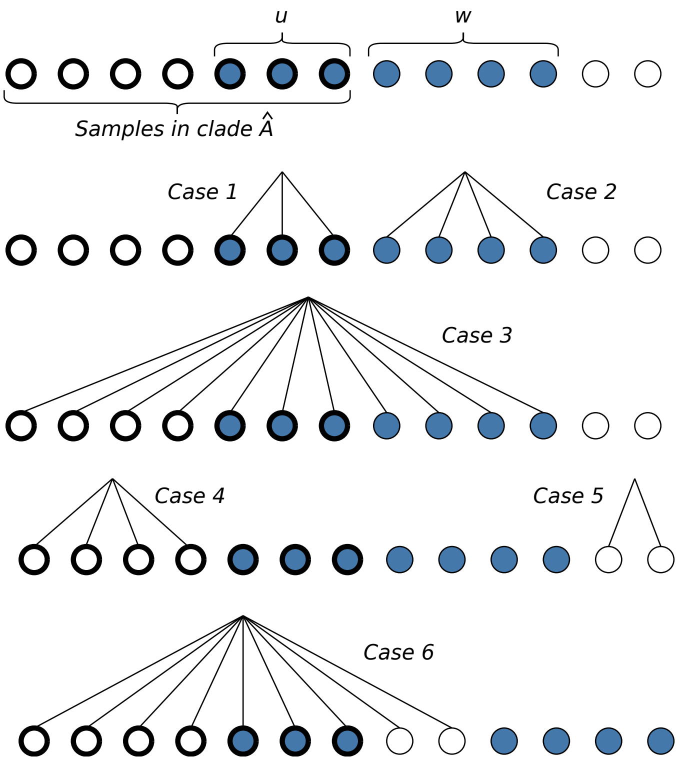

Figure S2: Samples of an ARG (ARG itself not shown) of size $n = 14$. Samples in clade $\widehat{A}$ shown with bold outline; samples in set S shown in blue. Lines connect samples that could possibly form a clade $\widehat{Z}$ in each considered case. Note that since we condition on A itself forming a clade, we cannot observe a clade formed of some samples from within $\widehat{A}$ and some samples from outside $\widehat{A}$ as this would violate that assumption (for instance, in the scenario shown, the set S itself cannot form a clade – only a set correlated with S can).

Taking each case in turn, for a fixed probability P, we calculate b from (S10), and take the minimum (and we repeat this procedure for each target set). Then, at each position of the genome, the maximum such value over all tested clades that span that position is informative of whether phantom epistasis can be effectively ruled out at that position. That is, we check whether the topology of the ARG implies that the samples in each target set are too well-separated for any problematic clade (one that is highly correlated with a target set) to be feasible. This is the basis for the procedure described in Methods, Section 2.4.2.

Note that, to limit the number of cases considered for each clade to six, we make a key simplifying assumption that Z being highly correlated with S is more likely to cause (S9) to be satisfied, which we expect to hold in practice unless S is small. Our simulation studies support this assumption: in cases of phantom epistasis, the mean correlation between the most significant explanatory clade and a target set was 0.32.

References

de los Campos, G., Sorensen, D. A., and Toro, M. A. Imperfect linkage disequilibrium generates phantom epistasis (& perils of big data). *G3: Genes, Genomes, Genetics*, **9**(5): 1429–1436, 2019.

Hein, J., Schierup, M., and Wiuf, C. *Gene genealogies, variation and evolution: A primer in coalescent theory*. Oxford University Press, USA, 2004.

Wooldridge, J. M. *Econometric analysis of cross section and panel data*. The MIT Press, Cambridge, Massachusetts, 2nd edn., 2010.

S2 Supplementary figures

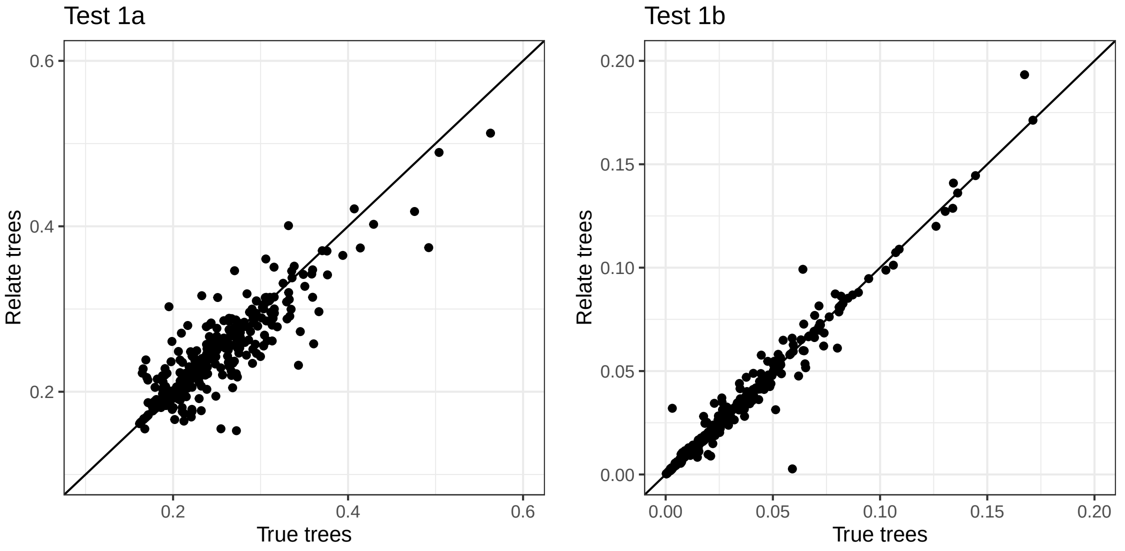

Figure S3: Values of b from Test 1A (left panel) and 1b (right panel) for true trees (x-axis) and Relate trees (y-axis), demonstrating close agreement.
